# The Assessment of Stent Effectiveness Using a Wearable Beamforming MEMS Microphone Array System

**DOI:** 10.1109/JTEHM.2016.2609901

**Published:** 2016-09-27

**Authors:** Metin Akay, Andrei Dragomir, Yasemin M. Akay, Feihua Chen, Allison Post, Hani Jneid, David Paniagua, Ali Denktas, Biykem Bozkurt

**Affiliations:** 1 Department of Biomedical EngineeringUniversity of Houston 14743 HoustonTX77204USA; 2 Winters Center for Heart Failure Research, DeBakey VA Medical CenterHoustonTX77030USA; 3 Cardiovascular Research Institute 233229 Baylor College of MedicineHoustonTX77030USA

**Keywords:** Coronary artery disease, nonlinear dynamics analysis, MEMs microphone array, approximate entropy

## Abstract

Studies involving turbulent flow have been carried out in many parts of the cardiovascular system, and it has been widely reported that turbulence related to stenosis (narrowing) of arteries creates audible sounds, which may be analyzed to yield information about the nature and severity of the blockage. Results so far indicate that the high frequency content of the sounds generally increases with the degree of stenosis. In this paper, we designed and built an MEMs microphone array and a signal acquisition board to improve the detection of coronary occlusions using an approach based on the recording and analysis of isolated diastolic heart sounds associated with turbulent blood flow in occluded coronary arteries. The nonlinear dynamic analysis method based on approximate entropy has been proposed for the analysis of diastolic heart sounds from patients with single coronary occlusions, before and after stent placement procedures. The nonlinear dynamic analysis (approximate entropy) measures of the diastolic heart sounds recorded from eight patients with single coronary occlusions and two normal subjects were estimated. In addition, a spectral analysis based on the fast Fourier transform was used to estimate the energy content of the recorded signals. Results suggest the presence of high nonlinear (approximate entropy) values of diastolic heart sounds associated with coronary artery disease ($p<0.01$) as well as significant differences in the energy content of the heart sound signals above and below 150 Hz ($p<0.05$).

## Graphical abstract

The goal of this research is to detect coronary occlusions using a noninvasive, passive, quick and inexpensive approach that could eventually be implemented as part of the standard medical exam. To improve the quality of the heart sound recordings associated with coronary occlusions, a novel MEMs microphone array platform was designed and used to record diastolic heart sounds from patients with coronary occlusions undergoing a coronary stent placement procedure. Results suggest the presence of more high frequency energy above 150Hz and high complexity values in diastolic heart sound signals of patients with coronary occlusions and significant decrease of these values after stent placement.

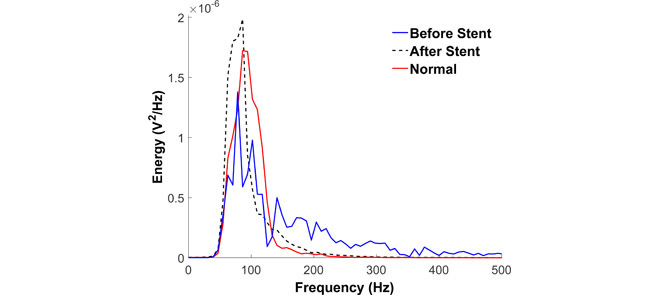

## I. Introduction

An estimated seven million Americans suffer from coronary artery disease (CAD), which causes more than half a million deaths annually and may cost more than $100 billion each year. For this reason, early detection of CAD is one of the most important research areas. Direct assessment of the coronary occlusion is usually done with cardiac catheterization and coronary angiography, followed by treatment using percutaneous coronary intervention (PCI) [1]–[3]. In conventional PCI, the occluding plaque is simply compressed and stented, and no material is removed. In almost one-third of cases, restenosis may occur within several months, which may require re-intervention. The first stent was used in 1986 and was developed in response to problems presented by the first angioplasties (performed in the previous decade, starting in 1977). However, of the coronary arteries that had been opened with a balloon, 30% would close, requiring a further angioplasty. A more permanent solution was needed.

In many cases, a stent was placed in the artery to keep the vessel open after angioplasty [4]. Despite the increased support to the artery wall provided by the stent, the risk of restenosis lingers, as plaque builds up around the stent, leading to fatal sub-acute thrombosis. It is also possible that the injury to the artery wall during balloon angioplasty causes increased smooth muscle growth.

Soon after the introduction of bare metal stents, drug eluting stents were approved by the FDA to reduce the occurrence of restenosis in the artery and decrease the risk of sub-acute thrombosis and latent sub-acute thrombosis, which can occur up to one year after the procedure [4]–[10]. However, there is still controversy surrounding the risks and benefits of drug eluting stents.

Non-invasive and lower risk modalities with varying degrees of accuracy and precision that have been proposed for diagnosis and risk stratification of CAD, such as coronary CT angiography, nuclear imaging and cardiac MRI, are still costly and associated with some risk. [1]–[3]. Alternative reliable, accurate and noninvasive approaches for early detection and monitoring of coronary obstructive lesions are limited.

### A. Turbulent Heart Sounds Associated With Coronary Occlusions

Initial studies showed that coronary arterial stenosis might give rise to diastolic murmurs [11]–[13]. Such diastolic murmurs are rarely heard, but can be a sign of a coronary stenosis. In 1967, it was first reported that the coronary stenosis could cause a diastolic murmur [12]. Three years later, two patients were reported with diastolic murmurs associated with a severe, localized narrowing of the left anterior descending (LAD) artery caused by coronary stenosis [14]. Three more years later, three cases with diastolic murmurs associated with CAD were also reported. For these cases, stenosis at the site of the murmur was proven by catheterization and the diastolic murmur was modified after surgery [15], [16]. Studies involving turbulent blood flow have been carried out for many components of the cardiovascular system and it has been widely reported that the turbulence produced by stenosis produces sounds due to the vibration of the surrounding structures [17]–[19]. These sounds have been detected and analyzed, and results generally showed that the high frequency energy increased with the degree of stenosis. However, for severe obstructions, above 95% occlusion, sounds may not be produced because of very low blood flow. At the lower end, occlusions as small as 25% in vessels of the neck, thorax and abdomen may produce sounds [20], [21].

In one of our previous studies, we investigated the effect of femoral artery stenosis on the turbulent sound generation mechanisms. We simulated an occluded artery by using a Teflon bead, which, after insertion into the femoral artery of a dog, created a reasonable simulation of sound generation in human coronary arteries [22]. The femoral artery of the dog was chosen since its dimensions and flow values are similar to those of human coronary arteries. Our in vitro testing indicated that the isolated sounds caused by occlusions contained more energy above 100 Hz than the background sounds recorded from vessels without occlusions [22].

In addition to the in vitro testing, Wang et al. [23] investigated the origin of turbulent sounds associated with occluded arteries using a sound source model, which combines an incremental network model of the left coronary artery tree with a transfer function model describing arterial chamber resonant characteristics. Their results suggested that the proposed model accurately predicts flow in both normal and occluded arteries.

In another previous study, we investigated the diagnostic capability of the acoustic approach in detecting CAD, and recorded heart sounds of 23 angioplasty patients before and after angioplastic surgery. Results from this angioplasty study showed that pre- and post-angioplasty records were correctly distinguished in 21 out of 23 cases [24], [25]. The spectral features most commonly associated with corrective coronary angioplasty consistently decreased at high frequency power (300–800 Hz). The relatively low frequency power (100–200 Hz) peak did not change with angioplasty and the amplitude of this peak was almost the same before and after coronary angioplasty. A parallel normal/abnormal study showed that the percentage of spectral energy above 300 Hz differed between normal and diseased patients, with the energy percentage over 300 Hz being greater in diseased subjects [25]–[27]. The results, using only the power spectral analysis characteristics, showed that 15 of 63 abnormal cases (a detection rate of 76%), and 6 of 37 (a false alarm of 16%) normal cases were incorrectly diagnosed. The angioplasty and normal/abnormal studies suggest that high frequency energy above 300 Hz is associated with coronary occlusions [25]. We also showed that sounds created by a controlled occlusion in the femoral artery of dogs were detected and analyzed by using the spectral analysis methods, including Fast Fourier Transform (FFT) and autoregressive methods [27]. The femoral vessel size and flow were chosen to approximate coronary artery conditions in patients. This work was done to confirm the hypothesis that coronary stenosis produces a detachable auditory correlation. The results confirm that high-frequency acoustic energy between 200 Hz and 800 Hz is associated with turbulence produced by stenosis [27].

Ye et al used the combination of wavelet transform and neural network methods to analyze the heart sounds recorded from 30 normal subjects and 30 subjects with coronary artery occlusions [28]. Their protocol allowed them to diagnose coronary artery occlusions, which showed a sensitivity of 86.7% [28]. Liang and Hartimo used a similar approach to report results based on the artificial neural network and wavelet transform methodologies [29]. Their results correctly classified about 75% of the heart murmurs, which included both systolic and diastolic murmurs [29]. In 1999, Borisyuk proposed an acoustic model of a larger human blood vessel [30]. Their model showed that as the degree of stenosis increases, the amount of turbulent flow and its associated sound additionally increases [30]. In 2001, Tateishi recorded and analyzed diastolic heart sounds from patients undergoing coronary angiography and from a control of young men [31]. Five piezoelectric sensors affixed to the skin were used to record from five different locations: three on the left sternal border, at the second, third and fourth intercostal spaces, and two on the right sternal border, at the second and third intercostal spaces. In this study, patients were divided into three categories based on their condition: stenosis group (50–75%), severe stenosis group (90–100%) and a normal group. Results suggested that, in terms of the relationship between the degree of stenosis and the sounds specific to the stenosis, there were significantly greater ($p<0.005$) power ratio values obtained in the stenosis group. Later, Banks et al. [32] developed a model suggesting that turbulent blood flow related to stenosis produces vibrations in the surrounding body tissue due to normal forces induced on the arterial wall [33]–[36].

In addition to the previous angioplasty and normal CAD studies, we recently introduced the nonlinear dynamic analysis method based on the approximate entropy and fractal estimators for the analysis of diastolic heart sounds associated with coronary occlusion [36]. The nonlinear dynamic analysis (approximate entropy and fractal) measures of diastolic heart sounds, recorded from 30 patients with coronary occlusions and 10 normal subjects, were estimated. The mean nonlinear dynamic analysis measures were calculated over 15 consecutive cardiac cycles. The mean approximate entropy measures were estimated for all abnormal and normal subjects and compared. The results suggest the presence of the high nonlinear (approximate entropy) values of diastolic heart sounds associated with coronary artery disease ($\text {p}<0.05$, one-way analysis of variance). This approach led to a sensitivity of 77%, a specificity of 80% and an overall accuracy of 78%. As a result, 23 out of 30 abnormal patients and 8 out of 10 normal patients were correctly detected [36]. We also used the FFT to analyze the same diastolic segments. This approach led to a sensitivity of 67% and a specificity of 70%, i.e. 20 out of 30 abnormal patients and 7 out of 10 normal patients were correctly detected [37]. The fractal analysis of the same database showed similar diagnostic performance to those of the approximate entropy.

In our previous studies, we used one sensor (accelerometer) or the commercially available e- stethoscope to detect the acoustical signals associated coronary occlusions. In this study, we designed an auscultation system based on a compact, ultra-low noise MEMS microphone array to improve the signal-to-noise ratio (SNR) and reduce electromagnetic interference (EMI). Microphone designs based on MEMS technology have become increasingly popular in the past two decades, with various applications in aerospace and military industry, as well as speech recognition [38]–[41], their most attractive features being their small size, lightweight, low power and low cost [42], [43]. Biomedical applications are primarily in the area of improving hearing aids performance [44], [45]. Designs based on arrays of sensors rely on the improved performance in terms of SNR compared to individual sensors [46]. Using our system, we have recorded and isolated diastolic heart sounds from CAD patients before and after stent placement, and applied nonlinear dynamic analysis and frequency domain analysis to investigate whether our system can be used to detect coronary occlusions and assess any differences resulting from the stent placement procedure. This is a preliminary proof-of-principle study intended to show the efficacy of the platform in clinics. Although it has been extremely challenging to find patients with only one occlusion, since almost all patients will have multiple occlusions with different levels of severity, it is crucial to show the changes in acoustic parameters before and after stent placement due to our ability to relate these changes to the modification of the stenosis.

## II. Methods

### A. Human Subjects

Subjects were recruited from a set of patients undergoing angioplasty surgery at the DeBakey VA Medical Center hospital. The DeBakey VA Hospital R&D Committee and Baylor College of Medicine IRB approved the study. Study staff members who had completed the human subject protection educational requirements in compliance with all Federal regulations conducted informed consent negotiations. Prospective subjects were interviewed to determine preliminary eligibility. Informed consent was obtained and subjects were enrolled in the study prior to any clinical testing, laboratory testing or intervention. Subjects were given a copy of the IRB approved consent form during the initial interview, and study staff explained to the subjects, in detail, the nature of the informed consent process, study purpose and procedures, time commitments, risks, potential benefits, treatment alternatives, rights as research participants, study staff contact information, confidentiality procedures, and arrangements for medical care provided in case of injury during the study. Subjects were given adequate time to consider their decision and encouraged to ask questions, both during the initial interview and throughout the study. Subjects were provided with a signed copy of the completed consent form.

Eight patients with coronary artery disease, as determined by coronary angiography, were screened for the study.

### B. Patient Inclusion and Exclusion Criteria

Patients were screened and tested at the Michael E. DeBakey Houston VA Hospital in collaboration with the Winter Center for Cardiology Research of the Baylor College of Medicine. The inclusion criteria for study patients were as follows: Body mass index (BMI) less than 30, single lesion CAD, and patient consent for the procedure.

Patients were not enrolled in the study if they had one or more of the following exclusion criteria: heart valve disease, multi-vessel CAD and implanted pacemaker or defibrillator. We enrolled patients with a low BMI in order to minimize the amount of fatty tissue between the heart and the electronic stethoscope. The fatty tissue could muffle and attenuate the sounds in the recording. We excluded patients with valve disease, as this can disrupt the duration and magnitude of the S1 and S2 heart sounds. Leaky valves could also allow for regurgitation, which would cause high frequency noise in the diastolic window and interfere with our analysis of coronary artery sounds. Similarly, implanted pacemakers and defibrillators create noise, which would be difficult to eliminate from recordings.

### C. Prototype Development

**FIGURE 1.**
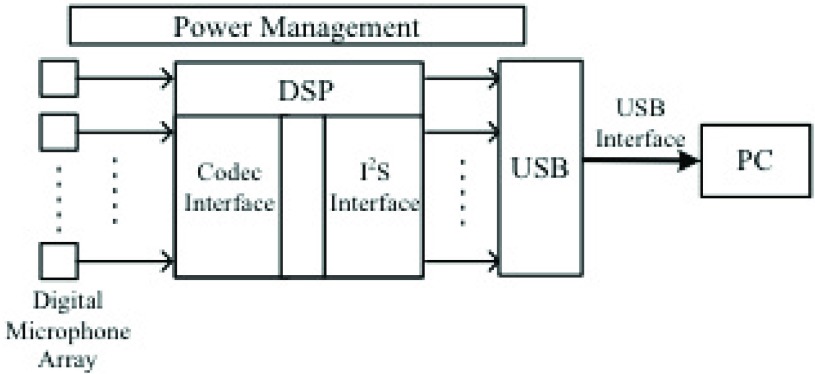
System architecture of our new compact system.

We built a compact MEMS microphone array to improve the signal-to-noise ratio (SNR) and reduce electromagnetic interference (EMI). This new system consisted of several modules, including the front-end sensor board (microphone array), the signal-processing unit for digital data acquisition and transfer, along with the interface to microphone array and the USB module for connection to a PC. Figure 1 shows the architecture of the new compact system.

There are several critical technical features in building the new system, including the design and integration of the front-end microphone array with high SNR, the multi-channel data acquisition and transfer and the design of the signal-processing unit and system integration.

**FIGURE 2.**
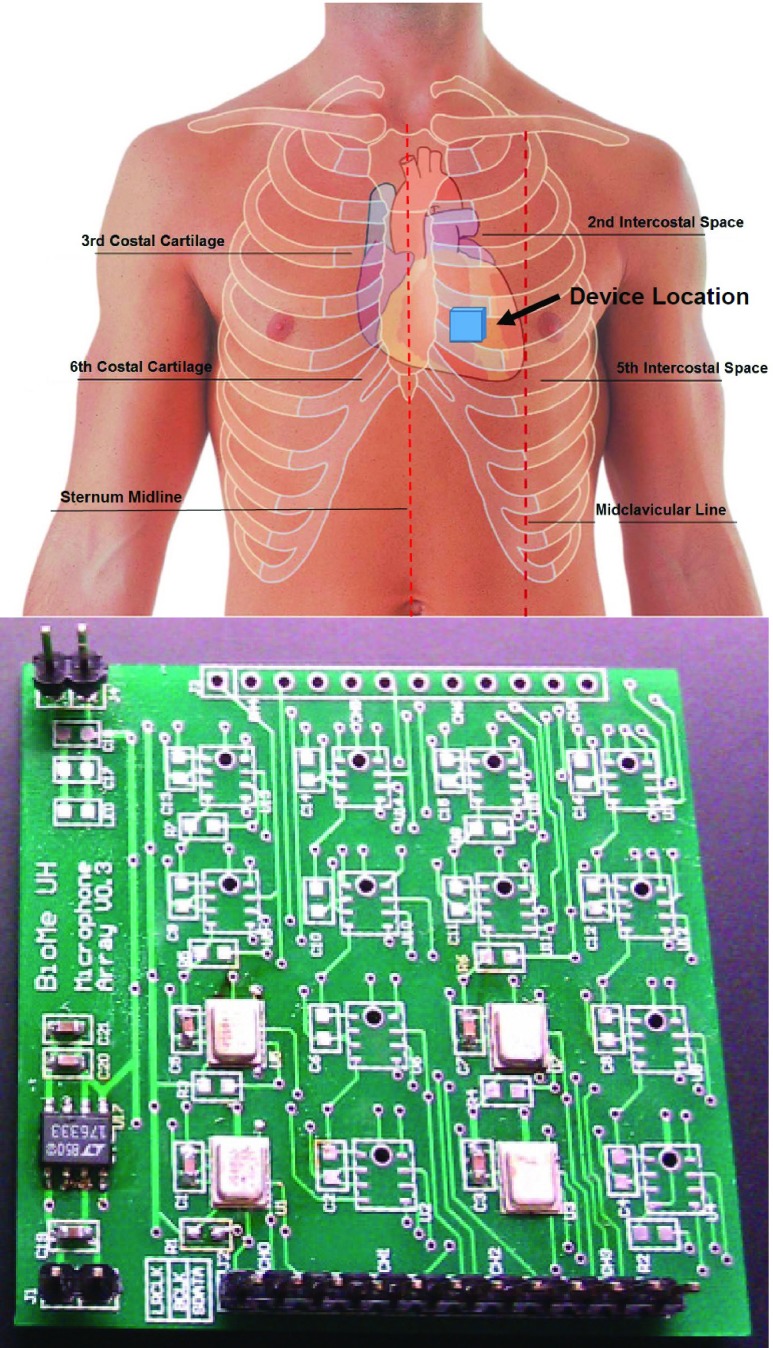
Overview of the recording location (top) and $2\times 2$ microphone array (bottom).

We have designed different configurations for the microphone array using digital microphones, such as several microphones placed in a line, circle, or rectangle. The digital microphone offers high SNR, flat frequency response and digital output with low power consumption and affordability. Our preliminary studies and lab tests showed that even two microphones improve the recording system performance, compared to that of a single microphone. Figure 2, lower panel, shows the picture of the $2\times 2$ microphone array. Each microphone on the array (ADI ADMP521) consists of a MEMS microphone element and an impedance converter amplifier followed by a fourth-order sigma-delta modulator. The digital interface allows for the pulse density modulated output of two microphones to be time-multiplexed on a single data line using a single clock. The microphones offer very high SNR (65dBA), sensitivity of ~26dBFS, extended wideband, flat frequency response and digital output with low current consumption ($900~\mu \text{A}$, and $< 1\mu \text{A}$ in sleep mode), compact size (4mm $\times3$mm $\times1$mm) and affordability [45].

The signal-processing unit handles data acquisition and transfer. The digital signal coming out of the microphone is received and rearranged by the signal-processing unit, then transferred to a PC through the USB (Figure 2, lower panel). As a result, the signal-processing unit should have interfaces to both the microphone array and the USB module. With this in mind, we designed our system to have a 4-channel interface for the whole unit. At the same time, the microphone interface is expandable in our design, as our aim is to integrate 30 channels into the system.

A Field Programmable Gate Array (FPGA) is used for the signal-processing unit design because of its programmability for multi-channel signal processing.

Digital data is transferred to a PC through the USB. The interface to the USB module is designed inside the FPGA. For portable consideration, a wireless module is under design using Wi-Fi for wireless data transfer. Our next version of the system will also integrate the wireless module. Figure 2, lower panel shows the $2\times 2$ channel microphone array and signal processing units.

### D. Heart Sounds Recordings From Patients

After finalizing our system, consisting of the microphone array unit and the signal-processing unit, we used the $2\times 2$ microphone array system at the VA cardiology unit to assess the effectiveness of stent placement in eight patients with single occlusion before and after the stent placement, as well as two subjects with clinically normal coronary arteries.

The array was placed at fourth intercostal space, 6–8 cm to the right of the midline of the sternum (Figure 2, top) and held in place for the duration of the recording. Sound was recorded for 15 seconds while the patient held his breath to reduce chest cavity noise. The patients were in the supine position and the recording device was held in place by its own weight in order to reduce motion artifacts related to manipulation.

**FIGURE 3.**
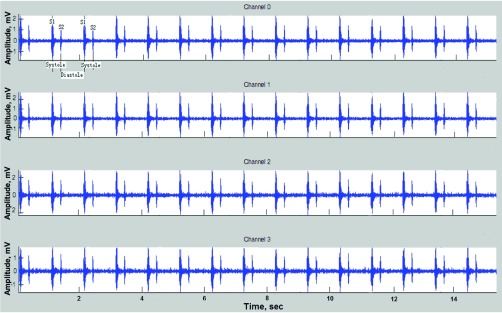
Four-channel raw recording from a healthy subject before beamforming.

Figure 3 shows the raw heart sound recordings from one normal subject using the 4-channel ($2\times 2$) prototype.

### E. Preprocessing

Raw 4-channel recordings were subsequently beamformed into a single signal using conventional time-delay beamforming, which is essentially a spatial filtering technique [46], [48]. Beamforming has been previously used in various biosignal processing applications, such as source localization, determining conduction velocity of motor unit action potentials, or as a multi sensor-based signal enhancement technique [49]–[52]. The idea behind time-delay beamforming is that when an acoustic signal is received by an array of microphones, it arrives with a certain amount of difference due to the distance from the sensor to the sound source. To compensate for arrival time differences, delays are introduced to each microphone output. The time-aligned signals are then summed together. This has the effect of enhancing wave components arriving from the source direction, while dampening off-axis noise, thus improving the SNR. The SNR of the beamformed signal is higher than those of individual microphones [46]. In our case, delays are easily estimated since we know the location of the source (Table 1) and the approximate speed of sound in human chest tissue ($\sim 1500\text{m}$/sec, [49]).

**TABLE 1:** Recruited Patient Information

Patient Number	Diseased Coronary	Percentage Stenosis
1	OM	90
2	Mid LAD	50
3	Left Main	70
4	Mid LAD	75
5	Mid LAD	95
6	Mid Cx	90
7	Mid Cx	80
8	Proximal LAD	95

Patient number and the corresponding coronary artery disease with percent stenosis. Abbreviations represent the following: Obtuse marginal(OM), Left Anterior descending (LAD), Circumflex (Cx)

Briefly, considering an array of $M$ microphones and $y_{m}$, the output of each microphone, the beamformed signal is computed by summing the signals as follows:

\begin{equation*} z\left ({ t }\right )=\sum \limits _{m=0}^{M-1} {y_{m}(t-\Delta _{m})} \end{equation*}

where $\Delta _{m}$ is the time delay between successive signals. In our implementation, we have used the phased.TimeDelay Beamformer and phased.URA objects of MATLAB’s Phased Array System Toolbox (Mathworks, Natick, Mass). The resulting beamformed signal was used for further processing and analysis.

SNR enhancement performance was evaluated by means of a gain measure computed as:

\begin{equation*} G=\frac {SNR_{o}}{SNR_{i}}, \end{equation*}

where SNR_*o*_ and SNR_*i*_ are the signal-to-noise ratios at the output and input of the beamformer. SNR_*i*_ was estimated as:

\begin{equation*} {SNR}_{i}=10\log \frac {\sigma _{y}^{2}-\sigma _{n}^{2}}{\sigma _{n}^{2}} \end{equation*}

where $\sigma _{y}^{2}$ and $\sigma _{n}^{2}$ are the recorded noisy signal and noise power, respectively. Noise power, $\sigma _{n}^{2}$, is estimated from the signal segments recorded after stent placement, as the stent placement procedure removes the signal of interest generated by the coronary occlusion. When calculating SNR_*i*_ the 4-channel powers are averaged into a single value. SNR_*o*_ is computed in a similar manner, using instead the beamformed signal to estimate the signal and noise powers, respectively.

### F. Diastolic Segment Selection

Having the second heart sound as a time reference, the diastolic heart sounds associated with coronary occlusions were preprocessed and 128 msec long segments located 100 msec after the second heart sound (S2) were isolated from the beamformed signal. The first (S1) and the second (S2) heart sound signals are clearly identified, as well as the diastolic and systolic segments. The recorded segments selected for analysis avoid the trailing edge of the second heart sound and the expected leading edge of the succeeding first heart sound, and coincide with the maximum coronary blood flow [25], [53].

All recordings were sampled at a rate of 4 kHz. Each isolated diastolic segment was initially normalized with respect to its mean energy value in order to account for sound attenuation effects due to physical differences between patients, such as chest mass and size. Isolated segments were then bandpass filtered between 65 and 500 Hz in order to reduce noise and because the amount of power above 500 Hz was negligible. The bandpass filter used was a Butterworth filter of order 5. Subsequently, segments were analyzed using the approximate entropy method and FFT-based spectral analysis.

### G. Approximate Entropy

The approximate entropy (ApEn) is a nonlinear measure quantifying the regularity of a time-series. ApEn is particularly efficient in the case of short data segments and is less sensitive to noise and outliers than other regularity statistics [54]. Additionally, it can be employed in the analysis of both stochastic and deterministic signals [54], [55]. This feature is particularly relevant in the case of biosignals, which are outputs of complex biological systems and may be deterministic, stochastic or both. ApEn provides a model-independent measure of the regularity of physical systems and physiological signals [55]–[58].

It characterizes a time series signal into a non-negative number, with higher ApEn values corresponding to more random signals, which generally reflect more complex generating systems. Basically, ApEn measures the logarithmic likelihood that runs of temporal patterns that are close within a certain threshold, $r$, over a defined number of observations, *m*, remain close in the next incremental comparisons. A higher likelihood for these segments of remaining close within the threshold produces smaller ApEn values, indicating a higher level of regularity for the signal.

In order to estimate approximate entropy values segments $X(i)$ through $X(N - m + 1)$ defined as $X(i) = \,\, [x(i), \ldots , x(i+ m - 1)]$ are considered. The difference between $X(i)$ and $X(j)$, $\text{d}[X(i), X(j)]$ can be computed as:

\begin{align*} d\left [{ X\left ({ i }\right ),X(j) }\right ]={max}_{k=0,m-1}\left [{ \left |{ x\left ({ i+k }\right )-x(j+k) }\right | }\right ]\leq r\notag \\ {}\end{align*}

assuming that all differences between the corresponding elements are less than the threshold $r$.

For any given $X(i)$, the ratio of the difference between $X(i)$ and $X(j)$ smaller than the threshold $r$ (denoted by $N_{r}^{m}$) to the total number of vectors ($N - m +1$) is obtained as:

\begin{equation*} C_{r}^{m}\left ({ i }\right )=\frac {N_{r}^{m}(i)}{N-m+1}, \quad \text {for}~ i = 1, \ldots , N-m +1 \end{equation*}

The approximate entropy, ApEn($m,r$), can be then estimated as:

\begin{align*} ApEn\left ({ m,r }\right )=&\lim \nolimits _{N\rightarrow \propto }\left [{ \Phi ^{m}\left ({ r }\right )-\Phi ^{m+1}\left ({ r }\right ) }\right ] \\ \text {with:}~\Phi ^{m}\left ({ r }\right )=&\sum \nolimits _{i=1}^{N-m+1} \ln {C_{r}^{m}\left ({ i }\right )} / {(N-m+1)}\qquad \end{align*}

In practice, the approximate entropy values can be estimated for a signal with $N$ samples as:

\begin{equation*} ApEn\left ({ m,r,N }\right )=\left [{ \Phi ^{m}\left ({ r }\right )-\Phi ^{m+1}\left ({ r }\right ) }\right ] \end{equation*}

Parameters $m$ and $r$ correspond to the embedding dimension of the analyzed signal and the threshold to suppress noise in the signal, respectively. For this study we have chosen $m =$ 2 as described in previous works [52]–[56]. The parameter $r$ can be chosen as $0.1\cdot \text {SD}(x)$, where SD($x$) represents the standard deviation of the original signal $x$.

## III. Results

We recorded and analyzed the diastolic heart sounds from eight patients with single occlusion and two clinically-proven normal subjects for approximately 15 heart cycles, as mentioned in the Methods section. The patients’ disease state is listed in Table 1.

**FIGURE 4.**
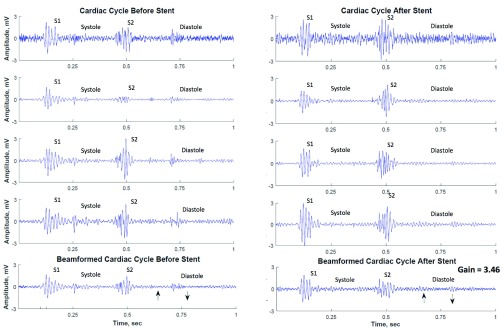
Cardiac cycle signal before (left panels) and after stent placement (right panels). The 4 channels (top four rows) correspond to recordings from the 4 microphones on the array.. Bottom row displays the beamformed signal obtained from the sensor array before and after stent placement (left and right column, respectively). Diastolic windows selected for the analysis are identified by arrows.

**FIGURE 5.**
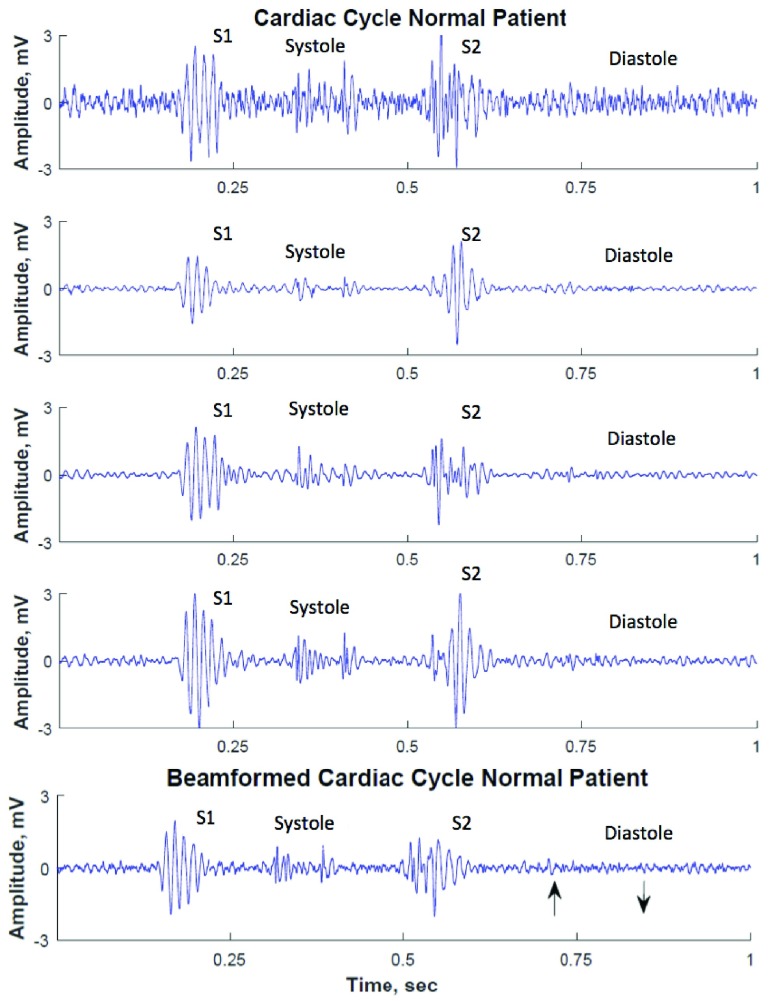
Single representative cardiac cycle of a normal subject with the diastolic segment identified. Top four channels correspond to recordings from the 4 microphones on the array. Bottom row displays the beamformed signal. Diastolic window selected for the analysis are identified by arrows.

**FIGURE 6.**
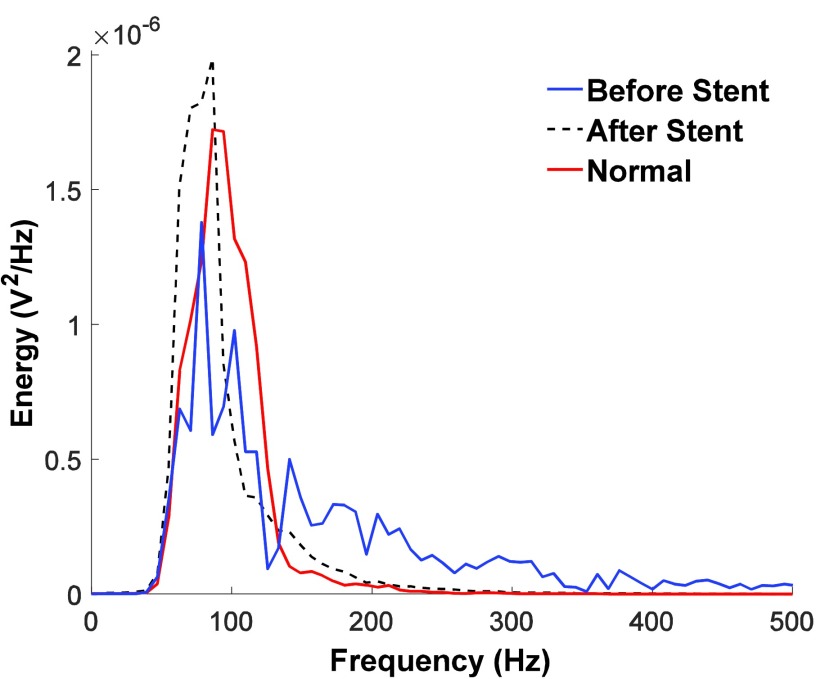
Power spectrum of the diastolic segments of the beamformed signals shown in the bottom panels of Figures 4 and 5.

We initially analyzed the diastolic segments of heart sounds using spectral analysis techniques based on FFT. Figure 4 shows the diastolic heart segments before and after stent placement in patient #4 (top 4 rows correspond to channels recorded by the microphone array and the bottom row displays the beamformed signal). The estimated beamformer SNR gain, $G$, for the data in Figure 4 is 3.46, while the average beamformer gain for all recordings was 2.81±0.38. The presence of relatively high amplitude signal is visible in the diastolic segments of recordings before stent placement, whereas diastolic segments after stent placement look more similar to those recorded from clinically normal patients (Figure 5). Figure 5 shows the diastolic segment in a clinically proven normal subject (4 channels recorded by the microphone array and the corresponding beamformed signal – data belongs to clinically normal patient #1) and Figure 6 shows the corresponding power spectra of the beamformed diastolic segments in Figures 4 and 5. We noted that the power spectral energy of the diastolic segment before stent placement seemed to present significant peaks both below and above 150 Hz. However, the power spectral energy content above 150 Hz significantly decreased after the stent placement. At the same time, the peak below 150 Hz was increased after the stent placement. Furthermore, the spectral features of the post-stent case and those of the clinically proven normal subject seemed to be the same.

In order to quantify these changes in the spectral content we defined for each patient a ratio of the amounts of energy as follows:

\begin{equation*} \textit {Ratio}= \frac {\textit {Energy above}~ 150~ Hz}{\textit {Energy below}~ 150 ~Hz} \end{equation*}

Healthy patients with normal coronary physiology have smooth blood flow through these arteries, and therefore should not exhibit high power in frequencies above 150 Hz in recordings. Healthy patients may serve as a baseline in future studies, and serve as an interesting comparison in ours. We would expect the ratios of healthy subjects’ recordings to be lower than the ratios of the pre-stent recordings of study patients.

Generally, the power ratio of the total power above 150Hz or below 150Hz showed a downward trend between pre- and post-stent recordings. Not only was there a decrease in the power of the high energy frequencies (150Hz or greater) from pre-stent to post-stent, there was also an increase in the low frequency energy (below 150Hz) in seven of the eight patients (except patient #2).

**FIGURE 7.**
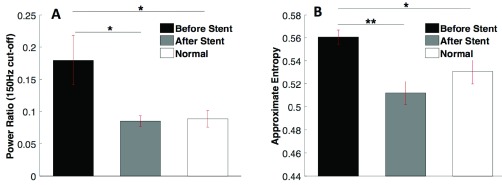
A) Comparison of all experimental group power ratios and B) approximate entropy values (** denotes $p<0.01$, * denotes $p<0.05$).

This increase in low frequency is most likely attributed to the improvement in heart function after stent placement. When blood flow is restored to the coronary artery, oxygen is again brought to the ischemic area of the heart. The heart muscle can then contribute to the contraction of the heart during the cardiac cycle, increasing the ejection fraction of the blood and therefore increasing the pressure on the valves when closing. The closing sounds may then become stronger, adding low frequency noise to the heart sound recording. All power ratio values were averaged across patients to give an average pre-stent ratio and an average post-stent ratio value (sample size: $n=80$). Ratios were compared using a one-way ANOVA statistical test as shown in Figure 7A. The power ratios for the diseased subjects before and after stent are significantly lower after stent placement ($p<0.05$). Power ratios after stent placement are not statistically different, however, from the clinically normal subjects.

As shown in Figure 7B, we obtained similar results on the same diastolic segments using the nonlinear dynamic analysis method based on the ApEn estimates. The average ApEn values significantly decreased after stent placement, except ($p<0.01$). The ApEn values after stent placement and those from normal subjects were not statistically significant. Patient 2 was the only patient who showed an increase in the power ratio and ApEn values. This is due to a heart murmur associated with a leaky valve. The heart murmur was not diagnosed until after the data was collected. Additionally, the lesion repaired was a borderline 50% stenosis, which could affect the analysis. Therefore, subject 2’s trend opposition is not unexpected.

As a summary, our approach demonstrates that the effectiveness of stent placement can be monitored using the BioMEMs based ($2\times 2$) microphone array.

## IV. Discussion and Conclusion

The ultimate goal of this research was to detect the presence and the location of coronary occlusions using a noninvasive, passive, quick and inexpensive approach that could eventually be implemented as part of the standard medical examination routine in the future. To improve the quality of the heart sound recordings, a novel, inexpensive MEMs microphone array platform was used to record diastolic heart sounds from CAD-diagnosed patients undergoing a coronary stent placement procedure, and normal patients without obstructive CAD.

Although the results of our previous studies, which applied the widely used power spectral analysis methods to recordings obtained using a single sensor (accelerometer or the electronic stethoscope), showed considerable promise, further improvements are needed for a reliable mass-screening procedure for the detection of coronary occlusions.

In this study, we used spectral analysis and a nonlinear dynamic analysis method to quantify and characterize the turbulent sounds associated with single coronary occlusions.

Overall, our results furthermore confirm the previous findings [16]–[20] and additionally show the usability of our MEMs microphone array in clinics to assess stent placement effectiveness. Our approach highlights the fact that CAD patients exhibit significantly different acoustic signatures in heart sounds recorded before and after stent placement procedures, and acoustic signatures of normal patients are similar to those recorded after stent placement. These findings indicate that our approach can be used for noninvasive continuous monitoring of CAD after stent placement.

This study has encouraged use to further develop and scale our platform for future longitudinal studies monitoring single or multiple in-stent restenosis as a low-cost and reliable surrogate for current imaging diagnostics preceding percutaneous coronary intervention. Our acoustical approach could be a low-cost noninvasive method to assess restenosis and/or development of *de novo* stenotic coronary lesions.

We plan to increase the patient enrollment in the next study. This study enrolled eight patients who underwent stent placement procedures with single occlusions, which is adequate for a preliminary efficacy study, despite the small sample size. For more robust results, future studies will include more patients, thus strengthening the statistics. Our first challenge was to find patients with only single coronary artery occlusions, as most patients with established coronary disease in our screening pool had multivessel disease. The second challenge was to have patients without any other cardiac disease. These two criteria limited the enrollment in our study.

For each subject, we would like to collect more recordings to increase the sample size for each subject; instead of simply 10 cardiac cycles for analysis, we could use 20 or 30 cycles. Additionally, we want to improve the recording quality, possibly by reducing the noise in the recording room. This would require patient isolation in a room with little to no electromagnetic interference.

Furthermore, implementation of a multichannel recording system would not only provide more data about the acoustic signature, it might also be used for ascertaining the location of the lesion. The beamforming technique can be successfully used to estimate the direction of arrival, as well as to localize the source of the heart sound (stenosis) when it is not known [41], [42]. We also plan to design and use a new $5\times 6$ wireless system to record diastolic heart sounds from 20 patients with multiple occlusions at the VA cardiology unit, and to follow the progression of coronary occlusions from these 20 patients by recording and analyzing their heart sounds before and after the stent placement, and again every 6 months over 5 years.

Finally, we plan to automate the recording analysis for clinical use. Instead of visually inspecting the signal and choosing the best sections by hand, we would create a program to perform these functions and calibrate it in another clinical study. Once the CAD acoustic signature – the power ratio – threshold has been optimized, the system could easily be implemented in a clinical setting, providing a simple, inexpensive and accurate screening tool for CAD.
